# Crystal structure of *catena*-poly[[aqua­bis­(4-formyl­benzoato)-κ^2^ *O* ^1^,*O* ^1′^;κ*O* ^1^-zinc]-μ-pyrazine-κ^2^ *N*:*N*′]

**DOI:** 10.1107/S2056989015005472

**Published:** 2015-03-21

**Authors:** Gülçin Şefiye Aşkın, Fatih Çelik, Nefise Dilek, Hacali Necefoğlu, Tuncer Hökelek

**Affiliations:** a Department of Physics, Hacettepe University, 06800 Beytepe, Ankara, Turkey; b Department of Chemistry, Kafkas University, 36100 Kars, Turkey; c Aksaray University, Department of Physics, 68100, Aksaray, Turkey

**Keywords:** crystal structure, zinc, transition metal complexes of benzoic acid derivatives, hydrogen bonding, π–π inter­actions, C—H⋯π inter­actions

## Abstract

The asymmetric unit of the title polymeric compound, [Zn(C_8_H_5_O_3_)_2_(C_4_H_4_N_2_)(H_2_O)]_*n*_, contains two mol­ecular units. Each unit comprises two 4-formyl­benzoate (FB) anions, one pyrazine mol­ecule and one coordinating water mol­ecule; the FB anions act either as bidentate or as monodentate ligands. The O atoms of the bidentately coordinating FB anions are disordered over two positions, and they were refined with fixed occupancy ratios of 0.75:0.25 and 0.70:0.30, respectively. In the ordered monodentately coordinating FB anions, the carboxyl­ate groups are twisted away from the attached benzene rings (*B* and *E*) by 12.1 (2) and 9.2 (2)°, respectively. In the disordered FB anions, the corresponding angles are 14.1 (1) and 4.0 (2)° for benzene rings *A* and *D*, respectively. Benzene rings *A* and *B* are oriented at a dihedral angle of 45.7 (1)°, *D* and *E* at 23.2 (1)°. Pyrazine ring *C* makes dihedral angles of 85.6 (1) and 72.7 (1)°, respectively, with benzene rings *A* and *B*, and pyrazine ring *F* makes dihedral angles of 87.0 (1) and 81.3 (1)° with benzene rings *D* and *E*, respectively. The pyrazine ligands bridge the Zn^II^ cations, forming polymeric chains running parallel to the *b*-axis direction. Medium-strength intra­molecular O—H⋯O hydrogen bonds link the water mol­ecules to the carboxyl­ate O atoms. In the crystal, water–carboxyl­ate O—H⋯O hydrogen bonds link adjacent chains into layers parallel to the *bc* plane. The layers are linked *via* weak pyrazine–formyl C—H⋯O and form­yl–carboxyl­ate C—H⋯O hydrogen bonds. π–π contacts between the benzene rings, with centroid-to-centroid distances of 3.7765 (16), 3.7905 (15) and 3.8231 (16) Å, may further stabilize the structure. There are also weak C—H⋯π inter­actions present.

## Abstract 2

In the compound, [Zn(C_8_H_5_O_3_)_2_(C_4_H_4_N_2_)(H_2_O)]_*n*_, the pyrazine ligands bridge the Zn^II^ cations, forming polymeric chains running parallel to the *b*-axis direction. Water–carboxyl­ate O—H⋯O hydrogen bonds link adjacent chains into layers parallel to the *bc* plane. The layers are linked *via* weak pyrazine–formyl C—H⋯O and form­yl–carboxyl­ate C—H⋯O hydrogen bonds.

## Chemical context   

The structural functions and coordination relationships of the aryl­carboxyl­ate ion in transition metal complexes of benzoic acid derivatives change depending on the nature and position of the substituent groups on the benzene ring, the nature of the additional ligand mol­ecule or solvent, and the medium of the synthesis (Adiwidjaja *et al.*, 1978 ▸; Antsyshkina *et al.*, 1980 ▸; Nadzhafov *et al.*, 1981 ▸; Shnulin *et al.*, 1981 ▸). Transition metal complexes with biochemically active ligands frequently show inter­esting physical and/or chemical properties, and as a result they may find applications in biological systems (Antolini *et al.*, 1982 ▸). Some benzoic acid derivatives, such as 4-amino­benzoic acid, have been extensively studied in coordination chemistry as bifunctional organic ligands due to their different coordination modes (Chen & Chen, 2002 ▸; Amiraslanov *et al.*, 1979 ▸; Hauptmann *et al.*, 2000 ▸).

In this context, we report the synthesis and crystal structure of the title compound, [Zn(C_8_H_5_O_3_)_2_(C_4_H_4_N_2_)(H_2_O)]_*n*_, which is closely related to its Cd analogue (Çelik *et al.*, 2014 ▸). In comparison with the latter, the title compound has a doubled *c* axis.
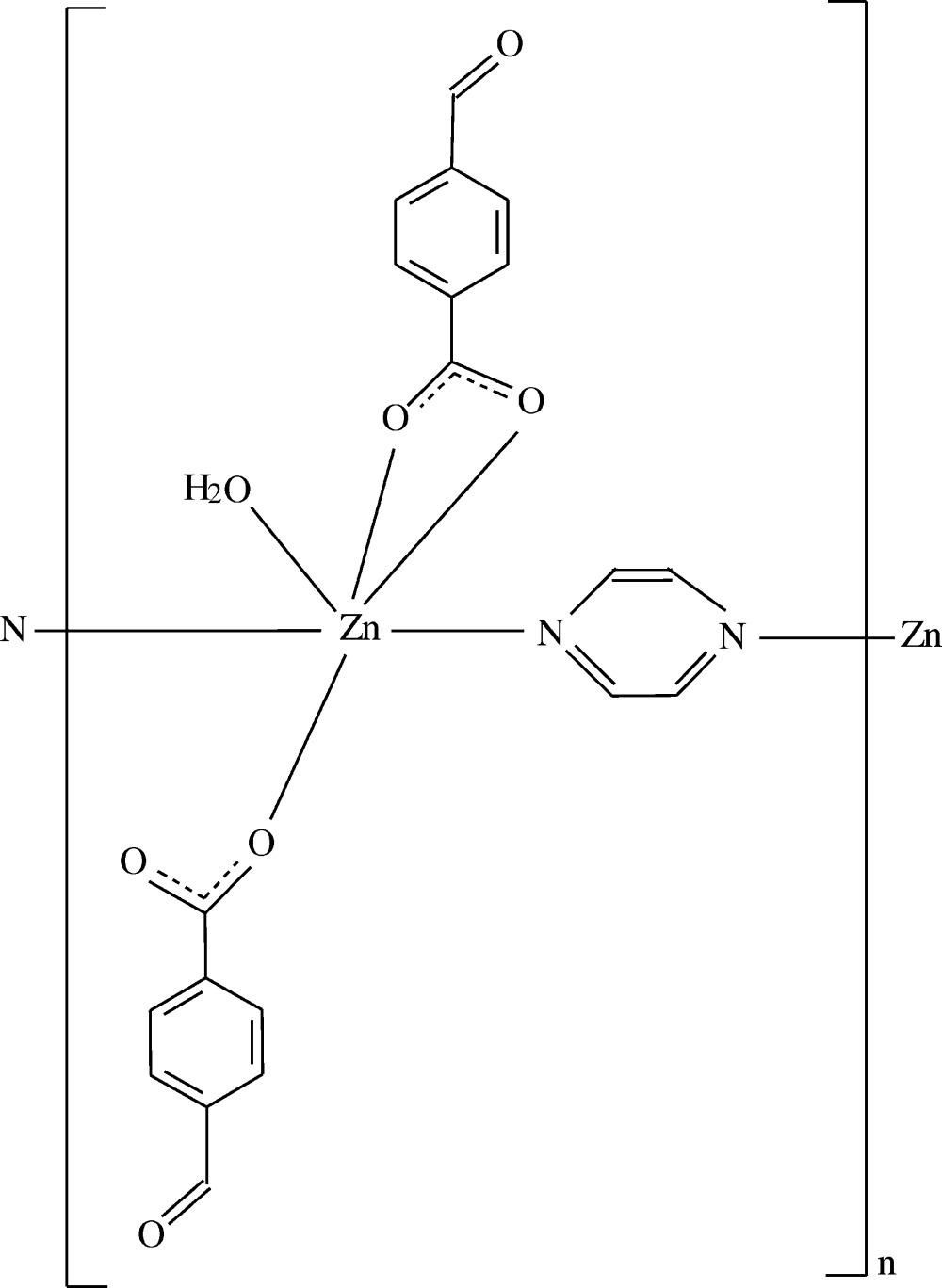


## Structural commentary   

**Figure 1:**
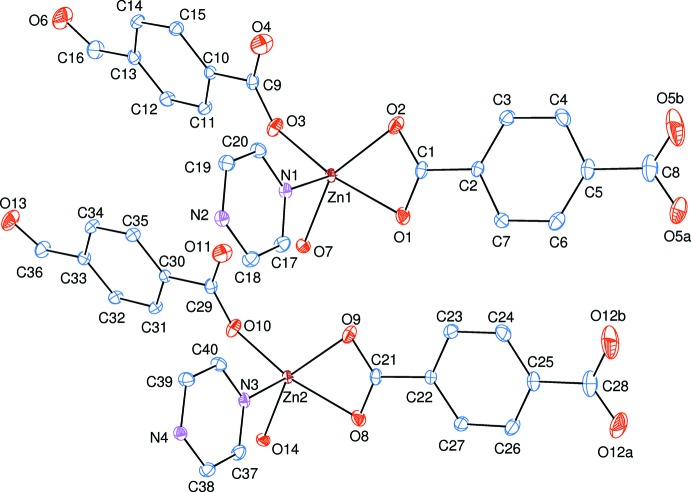
The asymmetric unit of the title compound, showing the atom-numbering scheme. Displacement ellipsoids are drawn at the 50% probability level. H atoms have been omitted for clarity and only the major occupancy components of the disordered carboxyl­ate O atoms are shown.

**Figure 2:**
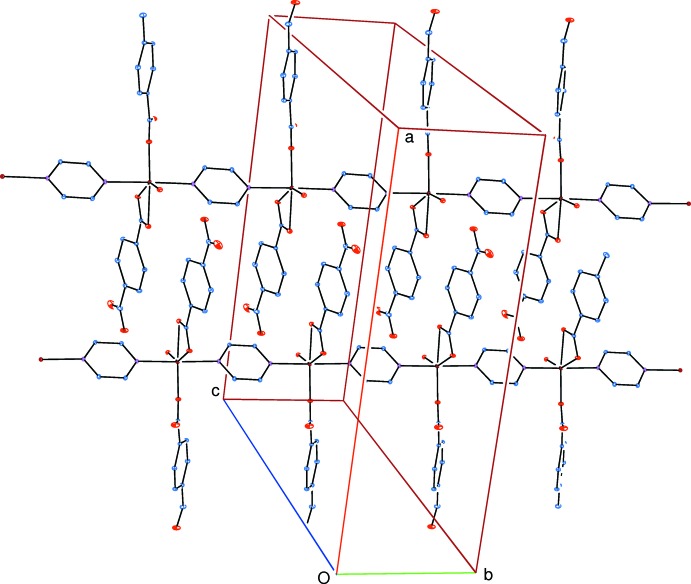
A partial view of the crystal packing of the title compound. H atoms have been omitted for clarity.

The asymmetric unit of the title polymeric compound contains two mol­ecular units. Each unit bears two 4-formyl­benzoate (FB) anions, one pyrazine mol­ecule and one coordinating water mol­ecule; the FB anions act either as bidentate or monodentate ligands (Fig. 1 ▸). The pyrazine ligands bridge adjacent Zn^II^ ions, forming polymeric chains running parallel to the *b*-axis direction (Fig. 2 ▸). The distances between the symmetry-related Zn^II^ ions [Zn1⋯Zn1^i^ and Zn2⋯Zn2^i^; symmetry code (i) *x*, *y* + 1, *z*] is 7.1729 (5) Å and corresponds to the length of the *b* axis.

The O1—Zn1—O2 and O8—Zn2—O9 angles are 58.88 (7) and 59.00 (7)°, respectively. The corresponding O—*M*—O (where *M* is a transition metal) angles are 52.91 (4) and 53.96 (4)° in [Cd(C_8_H_5_O_3_)_2_(C_6_H_6_N_2_O)_2_(H_2_O)]·H_2_O (Hökelek *et al.*, 2009 ▸), 53.50 (14)° in [Cu_2_(C_8_H_5_O_3_)_4_(C_6_H_6_N_2_O)_4_] (Sertçelik *et al.*, 2013 ▸) and 53.89 (17) and 53.88 (18)° in [Cd(C_8_H_5_O_3_)_2_(C_4_H_4_N_2_)(H_2_O)]_*n*_ (Çelik *et al.*, 2014 ▸).

The near equality of the C1—O1 [1.251 (3) Å], C1—O2 [1.256 (3) Å], C9—O3 [1.257 (3) Å], C9—O4 [1.227 (3) Å] and C21—O8 [1.248 (3) Å], C21—O9 [1.259 (3) Å], C29—O10 [1.258 (3) Å], C29—O11 [1.230 (3) Å] bonds in the carboxyl­ate groups indicate delocalized bonding arrangements, rather than localized single and double bonds. The average Zn—O and Zn—N distances are 2.11 (12) Å and 2.194 (6) Å, respectively, close to standard values. The Zn atoms lie 0.0484 (3) and 0.0571 (3) Å below [Zn1 relative to (O1/O2/C1) and (O3/O4/C9)] and 0.0623 (3) and 0.1322 (3) Å above [Zn2 relative to (O8/O9/C21) and (O10/O11/C29)] the carboxyl­ate groups. The dihedral angles between the planar carboxyl­ate groups [(O1/O2/C1), (O3/O4/C9) and (O8/O9/C21), (O10/O11/C29)] and the adjacent benzene rings [*A* (C2—C7), *B* (C10—C15) and *D* (C22—C27), *E* (C30—C35)] are 14.1 (2), 12.1 (2), 4.0 (2) and 9.2 (2)°, respectively, while the benzene rings are oriented at dihedral angles of 45.7 (1) and 23.2 (1)°. On the other hand, the pyrazine rings [*C* (N1/N2/C17–C20) and *F* (N3/N4/C37–C40)] are oriented at dihedral angles of 85.6 (1), 72.7 (1), 87.0 (1) and 81.3 (1)° with respect to benzene rings *A*, *B*, *D* and *E*, respectively.

## Supra­molecular features   

**Figure 3:**
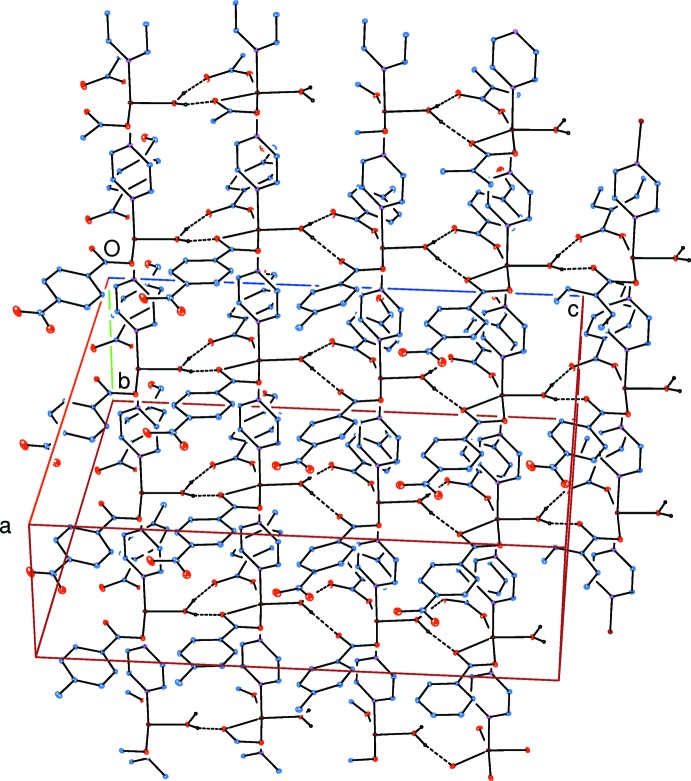
Part of the crystal structure. Inter­molecular water–carboxyl­ate O—H⋯O hydrogen bonds are shown as dashed lines. H atoms not involved in hydrogen bonds have been omitted for clarity.

**Figure 4:**
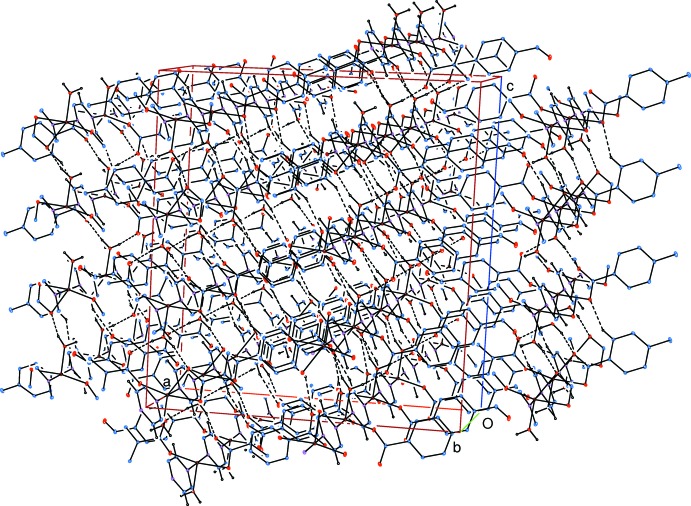
Part of the supra­molecular structure formed by the inter­molecular water–carboxyl­ate O—H⋯O, pyrazine–formyl C—H⋯O and form­yl–carboxyl­ate C—H⋯O hydrogen bonds. Hydrogen bonds are shown as dashed lines. H atoms not involved in hydrogen bonds have been omitted for clarity.

Medium-strength intra­molecular O—H⋯O hydrogen bonds (Table 1 ▸) link the water mol­ecules to the carboxyl­ate oxygen atoms. In the crystal, water–carboxyl­ate O—H⋯O hydrogen bonds (Table 1 ▸) link adjacent chains into layers parallel to the *bc* plane (Fig. 3 ▸). The layers are linked *via* pyrazine–formyl C—H⋯O and form­yl–carboxyl­ate C—H⋯O hydrogen bonds, forming a three-dimensional supra­molecular structure (Fig. 4 ▸). π–π contacts between the benzene rings, *A*⋯*A* ^i^, *B*⋯*B* ^ii^ and *D*⋯*D* ^iii^ with centroid-to-centroid distances of 3.7765 (16), 3.7905 (15) and 3.8231 (16) Å, respectively [symmetry codes: (i) 1 − *x*, −*y*, −*z*; (ii) −*x*, −*y*, −*z*; (iii) 1 − *x*, −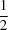 + *y*, 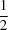 − *z*] may further stabilize the structure. There are also weak C—H⋯π inter­actions present (Table 1 ▸).

## Synthesis and crystallization   

The title compound was prepared by the reaction of ZnSO_4_·H_2_O (0.90 g, 5 mmol) in H_2_O (25 ml) and pyrazine (0.40 g, 5 mmol) in H_2_O (25 ml) with sodium 4-formyl­benzoate (1.72 g, 10 mmol) in H_2_O (70 ml). The mixture was filtered and set aside to crystallize at ambient temperature for one week, giving colorless single crystals.

## Refinement   

The experimental details including the crystal data, data collection and refinement are summarized in Table 2 ▸. Atoms H71, H72, H141, H142 (for H_2_O) and H16, H36 (for CH) were located in a difference Fourier map and the O7—H71, O7—H72, O14—H141, O14—H142, C16—H16, C36—H36 distances and H71—O7—H72 angle restrained to 0.897 (16), 0.866 (16), 0.826 (17), 0.845 (18), 0.943 (18), 0.937 (18) Å and 106 (2)°, respectively. The C-bound H atoms were positioned geometrically, with C—H = 0.93 and 0.98 Å for aromatic and methine H atoms, respectively, and constrained to ride on their parent atoms, with *U*_iso_(H) = 1.2*U*_eq_(C). The O atoms of the two bidentately coordinating FB anions are disordered over two positions. The O atoms (O5*A*, O5*B* and O12*A*, O12*B*) were refined with fixed occupancy ratios of 0.75:0.25 and 0.70:0.30, respectively.

## Supplementary Material

Crystal structure: contains datablock(s) I, global. DOI: 10.1107/S2056989015005472/wm5135sup1.cif

Structure factors: contains datablock(s) I. DOI: 10.1107/S2056989015005472/wm5135Isup2.hkl

CCDC reference: 1054503

Additional supporting information:  crystallographic information; 3D view; checkCIF report

## Figures and Tables

**Table 1:** Hydrogen-bond geometry (, ) *Cg*8 and *Cg*10 are the centroids of rings *B* (C10C15) and *E* (C30C35), respectively.

*D*H*A*	*D*H	H*A*	*D* *A*	*D*H*A*
O7H71O9	0.90(2)	1.82(2)	2.694(3)	165(2)
O7H72O11	0.87(2)	1.78(2)	2.640(3)	170(2)
O14H141O2^i^	0.83(2)	1.90(2)	2.705(3)	165(2)
O14H142O4^i^	0.84(2)	1.80(3)	2.635(3)	172(3)
C17H17O12*A* ^ii^	0.93	2.56	3.375(5)	146
C19H19O6^iii^	0.93	2.47	3.222(4)	138
C23H23O1	0.93	2.57	3.361(3)	143
C38H38O5*A* ^ii^	0.93	2.59	3.381(4)	144
C39H39O13^iv^	0.93	2.47	3.154(4)	130
C12H12*Cg*10^v^	0.93	2.81	3.579(3)	140
C32H32*Cg*8^v^	0.93	2.78	3.468(3)	132

Symmetry codes: (i) 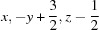; (ii) 
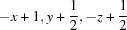
; (iii) 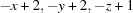; (iv) 
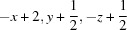
; (v) 
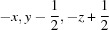
.

**Table 2:** Experimental details

Crystal data	
Chemical formula	[Zn(C_8_H_5_O_3_)_2_(C_4_H_4_N_2_)(H_2_O)]
*M*_r_	461.74
Crystal system, space group	Monoclinic, *P*2_1_/*c*
Temperature (K)	296
*a*, *b*, *c* ()	22.4721(7), 7.1729(2), 23.6377(8)
()	91.764(2)
*V* (^3^)	3808.4(2)
*Z*	8
Radiation type	Mo *K*
(mm^1^)	1.34
Crystal size (mm)	0.50 0.29 0.28

Data collection	
Diffractometer	Bruker SMART BREEZE CCD
Absorption correction	Multi-scan (*SADABS*; Bruker, 2012 ▸)
*T*_min_, *T*_max_	0.628, 0.676
No. of measured, independent and observed [*I* > 2(*I*)] reflections	87627, 9571, 7984
*R*_int_	0.031
(sin /)_max_ (^1^)	0.670

Refinement	
*R*[*F* ^2^ > 2(*F* ^2^)], *wR*(*F* ^2^), *S*	0.041, 0.102, 1.10
No. of reflections	9571
No. of parameters	583
No. of restraints	8
H-atom treatment	H atoms treated by a mixture of independent and constrained refinement
_max_, _min_ (e ^3^)	0.64, 0.65

Computer programs: *APEX2* and *SAINT* (Bruker, 2012 ▸), *SHELXS97* and *SHELXL97* (Sheldrick, 2008 ▸), *ORTEP-3* for Windows and *WinGX* (Farrugia, 2012 ▸) and *PLATON* (Spek, 2009 ▸).

## supplementary crystallographic information

### Crystal data

**Table:**

[Zn(C_8_H_5_O_3_)_2_(C_4_H_4_N_2_)(H_2_O)]	*F*(000) = 1888
*M**_r_* = 461.74	*D*_x_ = 1.611 Mg m^−^^3^
Monoclinic, *P*2_1_/*c*	Mo *K*α radiation, λ = 0.71073 Å
*a* = 22.4721 (7) Å	Cell parameters from 9153 reflections
*b* = 7.1729 (2) Å	θ = 2.7–28.3°
*c* = 23.6377 (8) Å	µ = 1.34 mm^−^^1^
β = 91.764 (2)°	*T* = 296 K
*V* = 3808.4 (2) Å^3^	Block, colorless
*Z* = 8	0.50 × 0.29 × 0.28 mm

### Data collection

**Table:**

Bruker SMART BREEZE CCD diffractometer	9571 independent reflections
Radiation source: fine-focus sealed tube	7984 reflections with *I* > 2σ(*I*)
Graphite monochromator	*R*_int_ = 0.031
φ and ω scans	θ_max_ = 28.4°, θ_min_ = 1.7°
Absorption correction: multi-scan (*SADABS*; Bruker, 2012)	*h* = −30→30
*T*_min_ = 0.628, *T*_max_ = 0.676	*k* = −9→9
87627 measured reflections	*l* = −31→31

### Refinement

**Table:**

Refinement on *F*^2^	Primary atom site location: structure-invariant direct methods
Least-squares matrix: full	Secondary atom site location: difference Fourier map
*R*[*F*^2^ > 2σ(*F*^2^)] = 0.041	Hydrogen site location: inferred from neighbouring sites
*wR*(*F*^2^) = 0.102	H atoms treated by a mixture of independent and constrained refinement
*S* = 1.10	*w* = 1/[σ^2^(*F*_o_^2^) + (0.0365*P*)^2^ + 4.5517*P*] where *P* = (*F*_o_^2^ + 2*F*_c_^2^)/3
9571 reflections	(Δ/σ)_max_ = 0.002
583 parameters	Δρ_max_ = 0.64 e Å^−^^3^
8 restraints	Δρ_min_ = −0.65 e Å^−^^3^

### Special details

**Table:**

Geometry. All esds (except the esd in the dihedral angle between two l.s. planes) are estimated using the full covariance matrix. The cell esds are taken into account individually in the estimation of esds in distances, angles and torsion angles; correlations between esds in cell parameters are only used when they are defined by crystal symmetry. An approximate (isotropic) treatment of cell esds is used for estimating esds involving l.s. planes.
Refinement. Refinement of F^2^ against ALL reflections. The weighted R-factor wR and goodness of fit S are based on F^2^, conventional R-factors R are based on F, with F set to zero for negative F^2^. The threshold expression of F^2^ > 2sigma(F^2^) is used only for calculating R-factors(gt) etc. and is not relevant to the choice of reflections for refinement. R-factors based on F^2^ are statistically about twice as large as those based on F, and R- factors based on ALL data will be even larger.

### Fractional atomic coordinates and isotropic or equivalent isotropic displacement parameters (Å^2^)

**Table:**

	*x*	*y*	*z*	*U*_iso_*/*U*_eq_	Occ. (<1)
Zn1	0.744994 (11)	0.75073 (4)	0.403123 (11)	0.02258 (7)	
Zn2	0.747592 (11)	0.65126 (4)	0.153050 (11)	0.02257 (7)	
O1	0.64782 (8)	0.7653 (3)	0.39486 (8)	0.0367 (4)	
O2	0.68812 (7)	0.7386 (3)	0.47961 (8)	0.0382 (4)	
O3	0.82950 (8)	0.7338 (3)	0.42612 (9)	0.0431 (5)	
O4	0.85759 (9)	0.8088 (4)	0.51393 (10)	0.0694 (8)	
O5A	0.36210 (12)	0.7654 (6)	0.51051 (16)	0.0724 (11)	0.75
O5B	0.3980 (6)	0.710 (2)	0.5801 (6)	0.107 (5)	0.25
O6	1.15317 (10)	0.6472 (5)	0.43755 (12)	0.0785 (9)	
O7	0.75486 (8)	0.7571 (3)	0.31819 (7)	0.0313 (4)	
H71	0.7277 (10)	0.721 (4)	0.2918 (10)	0.045 (9)*	
H72	0.7893 (8)	0.736 (4)	0.3040 (11)	0.033 (8)*	
O8	0.64987 (8)	0.6395 (3)	0.14295 (8)	0.0382 (4)	
O9	0.69021 (7)	0.6319 (3)	0.22812 (8)	0.0396 (4)	
O10	0.83197 (8)	0.6462 (3)	0.17805 (9)	0.0433 (5)	
O11	0.85687 (9)	0.7240 (4)	0.26661 (9)	0.0617 (7)	
O12A	0.36444 (14)	0.6022 (7)	0.25759 (18)	0.0804 (13)	0.70
O12B	0.4034 (4)	0.5896 (16)	0.3355 (5)	0.091 (3)	0.30
O13	1.15688 (9)	0.6937 (4)	0.18855 (11)	0.0677 (7)	
O14	0.75787 (8)	0.6458 (3)	0.06810 (8)	0.0330 (4)	
H141	0.7321 (10)	0.667 (4)	0.0432 (10)	0.031 (8)*	
H142	0.7912 (10)	0.665 (5)	0.0536 (13)	0.052 (10)*	
N1	0.74426 (8)	1.0554 (3)	0.40613 (8)	0.0266 (4)	
N2	0.74172 (8)	1.4441 (3)	0.40378 (8)	0.0272 (4)	
N3	0.74446 (8)	0.9567 (3)	0.15473 (9)	0.0273 (4)	
N4	0.74520 (8)	1.3452 (3)	0.15394 (8)	0.0261 (4)	
C1	0.64294 (10)	0.7548 (3)	0.44733 (11)	0.0287 (5)	
C2	0.58192 (10)	0.7557 (3)	0.47132 (10)	0.0262 (5)	
C3	0.57341 (11)	0.7017 (4)	0.52678 (11)	0.0337 (5)	
H3	0.6059	0.6714	0.5503	0.040*	
C4	0.51606 (12)	0.6931 (4)	0.54700 (11)	0.0373 (6)	
H4	0.5101	0.6543	0.5839	0.045*	
C5	0.46791 (11)	0.7419 (4)	0.51255 (13)	0.0376 (6)	
C6	0.47625 (11)	0.8009 (4)	0.45766 (12)	0.0396 (6)	
H6	0.4438	0.8366	0.4348	0.048*	
C7	0.53321 (11)	0.8062 (4)	0.43705 (11)	0.0324 (5)	
H7	0.5389	0.8440	0.4000	0.039*	
C8	0.40702 (14)	0.7343 (5)	0.53588 (17)	0.0566 (9)	
H8	0.4038	0.6009	0.5274	0.068*	
C9	0.86731 (10)	0.7533 (3)	0.46598 (11)	0.0307 (5)	
C10	0.93070 (10)	0.7064 (3)	0.45186 (10)	0.0257 (5)	
C11	0.94340 (11)	0.6169 (4)	0.40145 (11)	0.0316 (5)	
H11	0.9128	0.5835	0.3761	0.038*	
C12	1.00211 (11)	0.5777 (4)	0.38922 (11)	0.0349 (6)	
H12	1.0107	0.5146	0.3561	0.042*	
C13	1.04793 (11)	0.6319 (4)	0.42600 (11)	0.0331 (5)	
C14	1.03533 (11)	0.7237 (4)	0.47596 (11)	0.0349 (6)	
H14	1.0661	0.7617	0.5005	0.042*	
C15	0.97691 (11)	0.7583 (4)	0.48905 (11)	0.0319 (5)	
H15	0.9684	0.8167	0.5230	0.038*	
C16	1.11037 (13)	0.5922 (5)	0.41156 (15)	0.0518 (8)	
H16	1.1144 (15)	0.523 (5)	0.3781 (10)	0.064 (11)*	
C17	0.71703 (11)	1.1488 (3)	0.36395 (11)	0.0333 (5)	
H17	0.6983	1.0825	0.3347	0.040*	
C18	0.71592 (11)	1.3417 (3)	0.36259 (11)	0.0328 (5)	
H18	0.6968	1.4016	0.3323	0.039*	
C19	0.76825 (12)	1.3503 (3)	0.44611 (11)	0.0324 (5)	
H19	0.7863	1.4165	0.4758	0.039*	
C20	0.76983 (11)	1.1575 (3)	0.44727 (11)	0.0309 (5)	
H20	0.7892	1.0976	0.4775	0.037*	
C21	0.64505 (10)	0.6280 (3)	0.19529 (11)	0.0292 (5)	
C22	0.58421 (10)	0.6087 (3)	0.21928 (10)	0.0256 (5)	
C23	0.57669 (11)	0.6078 (4)	0.27715 (11)	0.0362 (6)	
H23	0.6096	0.6147	0.3019	0.043*	
C24	0.51980 (12)	0.5964 (4)	0.29793 (12)	0.0411 (6)	
H24	0.5147	0.5928	0.3368	0.049*	
C25	0.47064 (11)	0.5905 (4)	0.26150 (12)	0.0368 (6)	
C26	0.47806 (11)	0.5890 (4)	0.20375 (12)	0.0419 (7)	
H26	0.4451	0.5832	0.1790	0.050*	
C27	0.53487 (11)	0.5963 (4)	0.18292 (11)	0.0349 (6)	
H27	0.5400	0.5928	0.1440	0.042*	
C28	0.40987 (14)	0.5920 (5)	0.28524 (17)	0.0579 (9)	
H28	0.4083	0.4555	0.2839	0.069*	
C29	0.86873 (10)	0.6787 (3)	0.21806 (11)	0.0306 (5)	
C30	0.93360 (10)	0.6650 (3)	0.20327 (10)	0.0248 (5)	
C31	0.94955 (10)	0.5928 (3)	0.15123 (10)	0.0297 (5)	
H31	0.9204	0.5538	0.1251	0.036*	
C32	1.00910 (10)	0.5795 (4)	0.13868 (10)	0.0315 (5)	
H32	1.0200	0.5284	0.1043	0.038*	
C33	1.05277 (10)	0.6418 (4)	0.17684 (11)	0.0297 (5)	
C34	1.03676 (11)	0.7148 (4)	0.22858 (11)	0.0322 (5)	
H34	1.0660	0.7570	0.2542	0.039*	
C35	0.97750 (10)	0.7249 (3)	0.24191 (10)	0.0296 (5)	
H35	0.9669	0.7719	0.2768	0.035*	
C36	1.11601 (12)	0.6292 (5)	0.16198 (13)	0.0442 (7)	
H36	1.1225 (14)	0.564 (4)	0.1284 (10)	0.055 (10)*	
C37	0.71896 (11)	1.0551 (4)	0.11238 (11)	0.0335 (5)	
H37	0.7006	0.9920	0.0822	0.040*	
C38	0.71914 (12)	1.2480 (3)	0.11217 (11)	0.0335 (6)	
H38	0.7006	1.3111	0.0821	0.040*	
C39	0.77052 (12)	1.2490 (3)	0.19636 (11)	0.0328 (5)	
H39	0.7890	1.3126	0.2264	0.039*	
C40	0.76986 (11)	1.0558 (3)	0.19668 (11)	0.0321 (5)	
H40	0.7877	0.9932	0.2272	0.039*	

### Atomic displacement parameters (Å^2^)

**Table:**

	*U*^11^	*U*^22^	*U*^33^	*U*^12^	*U*^13^	*U*^23^
Zn1	0.02006 (13)	0.02144 (13)	0.02640 (14)	0.00033 (9)	0.00340 (10)	−0.00053 (10)
Zn2	0.02074 (13)	0.02083 (13)	0.02630 (14)	−0.00049 (10)	0.00335 (10)	0.00102 (10)
O1	0.0300 (9)	0.0457 (11)	0.0349 (10)	−0.0038 (8)	0.0098 (8)	−0.0034 (8)
O2	0.0223 (8)	0.0499 (12)	0.0423 (11)	0.0011 (8)	−0.0006 (7)	−0.0106 (9)
O3	0.0236 (9)	0.0478 (12)	0.0574 (13)	0.0031 (8)	−0.0049 (8)	−0.0026 (10)
O4	0.0330 (11)	0.127 (2)	0.0487 (14)	0.0129 (13)	0.0135 (10)	−0.0189 (15)
O5A	0.0228 (14)	0.105 (3)	0.090 (3)	0.0029 (16)	0.0116 (15)	0.006 (2)
O5B	0.083 (9)	0.128 (12)	0.114 (11)	−0.029 (8)	0.070 (8)	−0.022 (9)
O6	0.0254 (11)	0.123 (3)	0.088 (2)	0.0033 (13)	0.0009 (12)	−0.0048 (18)
O7	0.0287 (9)	0.0424 (11)	0.0229 (9)	−0.0004 (8)	0.0060 (7)	−0.0031 (8)
O8	0.0317 (9)	0.0470 (11)	0.0365 (10)	0.0034 (8)	0.0115 (8)	0.0083 (9)
O9	0.0229 (8)	0.0515 (12)	0.0444 (11)	−0.0041 (8)	0.0007 (8)	−0.0131 (9)
O10	0.0234 (8)	0.0470 (12)	0.0592 (13)	0.0007 (8)	−0.0036 (8)	−0.0072 (10)
O11	0.0315 (10)	0.113 (2)	0.0416 (12)	0.0092 (12)	0.0132 (9)	−0.0065 (13)
O12A	0.0252 (16)	0.127 (4)	0.090 (3)	0.0002 (19)	0.0110 (17)	−0.001 (3)
O12B	0.065 (6)	0.109 (8)	0.102 (8)	−0.022 (5)	0.055 (6)	−0.029 (6)
O13	0.0263 (10)	0.102 (2)	0.0753 (17)	−0.0053 (12)	0.0011 (11)	−0.0160 (15)
O14	0.0289 (9)	0.0440 (11)	0.0261 (9)	0.0013 (8)	0.0036 (8)	0.0062 (8)
N1	0.0242 (9)	0.0219 (9)	0.0339 (11)	0.0006 (8)	0.0043 (8)	0.0010 (8)
N2	0.0276 (9)	0.0213 (9)	0.0328 (11)	0.0025 (8)	0.0033 (8)	0.0017 (8)
N3	0.0241 (9)	0.0202 (9)	0.0378 (11)	−0.0009 (8)	0.0056 (8)	0.0024 (8)
N4	0.0279 (10)	0.0212 (9)	0.0293 (10)	0.0006 (8)	0.0015 (8)	0.0019 (8)
C1	0.0233 (11)	0.0258 (12)	0.0372 (14)	−0.0035 (9)	0.0057 (10)	−0.0090 (10)
C2	0.0207 (10)	0.0272 (12)	0.0308 (12)	−0.0009 (9)	0.0033 (9)	−0.0050 (9)
C3	0.0276 (12)	0.0425 (14)	0.0309 (13)	0.0020 (11)	−0.0002 (10)	−0.0018 (11)
C4	0.0385 (14)	0.0435 (15)	0.0305 (14)	−0.0034 (12)	0.0127 (11)	−0.0022 (11)
C5	0.0273 (12)	0.0373 (14)	0.0487 (16)	−0.0041 (10)	0.0113 (11)	−0.0088 (12)
C6	0.0244 (12)	0.0466 (16)	0.0475 (17)	0.0026 (11)	−0.0026 (11)	−0.0021 (13)
C7	0.0292 (12)	0.0385 (14)	0.0294 (13)	0.0000 (10)	0.0015 (10)	0.0041 (11)
C8	0.0388 (17)	0.057 (2)	0.076 (3)	−0.0081 (15)	0.0245 (17)	−0.0156 (18)
C9	0.0214 (11)	0.0299 (13)	0.0410 (14)	0.0004 (9)	0.0052 (10)	0.0038 (11)
C10	0.0219 (10)	0.0270 (11)	0.0284 (12)	0.0012 (9)	0.0027 (9)	0.0028 (9)
C11	0.0277 (11)	0.0367 (13)	0.0302 (13)	−0.0012 (10)	−0.0025 (10)	−0.0047 (10)
C12	0.0346 (13)	0.0396 (14)	0.0306 (13)	0.0051 (11)	0.0055 (10)	−0.0060 (11)
C13	0.0247 (11)	0.0368 (13)	0.0381 (14)	0.0026 (10)	0.0051 (10)	0.0052 (11)
C14	0.0233 (11)	0.0467 (15)	0.0345 (14)	−0.0022 (11)	−0.0042 (10)	0.0007 (12)
C15	0.0285 (12)	0.0397 (14)	0.0275 (13)	−0.0003 (10)	0.0015 (10)	−0.0044 (10)
C16	0.0300 (14)	0.070 (2)	0.056 (2)	0.0089 (14)	0.0085 (14)	0.0000 (17)
C17	0.0341 (13)	0.0259 (12)	0.0392 (14)	0.0006 (10)	−0.0065 (11)	−0.0035 (10)
C18	0.0335 (12)	0.0260 (12)	0.0386 (14)	0.0046 (10)	−0.0065 (11)	0.0022 (10)
C19	0.0391 (13)	0.0257 (12)	0.0323 (13)	−0.0029 (10)	−0.0014 (11)	−0.0017 (10)
C20	0.0346 (12)	0.0262 (12)	0.0319 (13)	0.0012 (10)	0.0002 (10)	0.0044 (10)
C21	0.0239 (11)	0.0220 (11)	0.0421 (15)	−0.0005 (9)	0.0064 (10)	−0.0019 (10)
C22	0.0229 (10)	0.0243 (11)	0.0297 (12)	−0.0017 (8)	0.0034 (9)	−0.0015 (9)
C23	0.0309 (12)	0.0489 (16)	0.0288 (13)	−0.0028 (11)	−0.0006 (10)	−0.0011 (11)
C24	0.0422 (15)	0.0528 (17)	0.0289 (14)	−0.0046 (13)	0.0115 (11)	−0.0025 (12)
C25	0.0288 (12)	0.0343 (14)	0.0481 (16)	−0.0030 (10)	0.0127 (11)	−0.0048 (12)
C26	0.0260 (12)	0.0557 (18)	0.0438 (16)	−0.0033 (12)	−0.0030 (11)	−0.0059 (13)
C27	0.0305 (12)	0.0480 (16)	0.0263 (13)	−0.0023 (11)	0.0012 (10)	−0.0029 (11)
C28	0.0391 (17)	0.054 (2)	0.082 (3)	−0.0064 (14)	0.0261 (17)	−0.0099 (18)
C29	0.0231 (11)	0.0282 (12)	0.0407 (14)	0.0024 (9)	0.0048 (10)	0.0026 (10)
C30	0.0223 (10)	0.0234 (11)	0.0287 (12)	0.0027 (8)	0.0031 (9)	0.0021 (9)
C31	0.0267 (11)	0.0333 (13)	0.0289 (12)	0.0014 (10)	−0.0036 (9)	−0.0064 (10)
C32	0.0304 (12)	0.0380 (14)	0.0263 (12)	0.0051 (10)	0.0024 (9)	−0.0068 (10)
C33	0.0244 (11)	0.0326 (13)	0.0321 (13)	0.0033 (9)	0.0042 (9)	−0.0007 (10)
C34	0.0260 (11)	0.0367 (13)	0.0337 (14)	0.0007 (10)	−0.0044 (10)	−0.0076 (11)
C35	0.0290 (12)	0.0326 (13)	0.0272 (12)	0.0046 (10)	0.0026 (9)	−0.0075 (10)
C36	0.0272 (13)	0.0592 (19)	0.0465 (17)	0.0049 (13)	0.0065 (12)	−0.0079 (15)
C37	0.0323 (12)	0.0257 (12)	0.0420 (14)	−0.0025 (10)	−0.0074 (11)	−0.0023 (11)
C38	0.0357 (13)	0.0265 (12)	0.0378 (14)	0.0007 (10)	−0.0079 (11)	0.0031 (10)
C39	0.0423 (14)	0.0256 (12)	0.0302 (13)	0.0001 (10)	−0.0038 (11)	0.0004 (10)
C40	0.0398 (13)	0.0253 (12)	0.0312 (13)	0.0029 (10)	−0.0002 (10)	0.0054 (10)

### Geometric parameters (Å, º)

**Table:**

Zn1—O1	2.1889 (18)	C10—C9	1.511 (3)
Zn1—O2	2.2477 (19)	C10—C15	1.390 (3)
Zn1—O3	1.9628 (18)	C11—C10	1.391 (3)
Zn1—O7	2.0271 (17)	C11—C12	1.388 (3)
Zn2—O8	2.2034 (18)	C11—H11	0.9300
Zn2—O9	2.2297 (19)	C12—C13	1.383 (4)
Zn2—O10	1.9689 (18)	C12—H12	0.9300
Zn2—O14	2.0288 (18)	C13—C16	1.482 (4)
Zn1—N1	2.186 (2)	C14—C13	1.389 (4)
Zn1—N2^i^	2.200 (2)	C14—H14	0.9300
Zn2—N3	2.192 (2)	C15—C14	1.380 (3)
Zn2—N4^i^	2.1957 (19)	C15—H15	0.9300
Zn1—C1	2.550 (2)	C16—H16	0.943 (18)
Zn2—C21	2.545 (2)	C17—H17	0.9300
O1—C1	1.251 (3)	C18—C17	1.384 (3)
O2—C1	1.256 (3)	C18—H18	0.9300
O3—C9	1.257 (3)	C19—H19	0.9300
O4—C9	1.227 (3)	C21—C22	1.503 (3)
O5A—C8	1.179 (5)	C22—C23	1.383 (3)
O6—C16	1.192 (4)	C22—C27	1.385 (3)
O7—H71	0.897 (16)	C23—C24	1.386 (4)
O7—H72	0.866 (16)	C23—H23	0.9300
O8—C21	1.248 (3)	C24—H24	0.9300
O9—C21	1.259 (3)	C25—C24	1.380 (4)
O10—C29	1.258 (3)	C25—C28	1.492 (4)
O11—C29	1.230 (3)	C26—C25	1.380 (4)
O13—C36	1.190 (4)	C26—H26	0.9300
O14—H141	0.826 (17)	C27—C26	1.383 (3)
O14—H142	0.845 (18)	C27—H27	0.9300
N1—C17	1.334 (3)	C28—O12A	1.197 (5)
N1—C20	1.333 (3)	C28—O12B	1.202 (10)
N2—Zn1^ii^	2.200 (2)	C28—H28	0.9800
N2—C18	1.338 (3)	C31—C32	1.383 (3)
N2—C19	1.331 (3)	C31—C30	1.392 (3)
N3—C37	1.339 (3)	C31—H31	0.9300
N3—C40	1.334 (3)	C34—C35	1.380 (3)
N4—Zn2^ii^	2.1957 (19)	C34—C33	1.388 (3)
N4—C38	1.330 (3)	C34—H34	0.9300
N4—C39	1.331 (3)	C20—C19	1.384 (3)
C2—C1	1.500 (3)	C20—H20	0.9300
C2—C3	1.386 (3)	C30—C35	1.392 (3)
C2—C7	1.390 (3)	C30—C29	1.513 (3)
C3—C4	1.390 (3)	C32—C33	1.386 (3)
C3—H3	0.9300	C32—H32	0.9300
C4—H4	0.9300	C33—C36	1.477 (3)
C5—C4	1.379 (4)	C35—H35	0.9300
C5—C6	1.383 (4)	C36—H36	0.937 (18)
C5—C8	1.492 (4)	C37—H37	0.9300
C6—H6	0.9300	C38—C37	1.383 (3)
C7—C6	1.384 (3)	C38—H38	0.9300
C7—H7	0.9300	C39—H39	0.9300
C8—O5B	1.086 (14)	C40—C39	1.386 (3)
C8—H8	0.9800	C40—H40	0.9300
			
O1—Zn1—O2	58.88 (7)	C11—C10—C9	120.8 (2)
O1—Zn1—N2^i^	90.85 (7)	C15—C10—C9	119.5 (2)
O1—Zn1—C1	29.37 (7)	C15—C10—C11	119.8 (2)
O2—Zn1—C1	29.52 (7)	C10—C11—H11	120.2
O3—Zn1—O1	169.01 (8)	C12—C11—C10	119.6 (2)
O3—Zn1—O2	110.13 (8)	C12—C11—H11	120.2
O3—Zn1—O7	98.12 (8)	C11—C12—H12	119.8
O3—Zn1—N1	93.49 (8)	C13—C12—C11	120.4 (2)
O3—Zn1—N2^i^	88.20 (8)	C13—C12—H12	119.8
O3—Zn1—C1	139.64 (9)	C12—C13—C14	120.0 (2)
O7—Zn1—O1	92.86 (7)	C12—C13—C16	119.5 (3)
O7—Zn1—O2	151.61 (7)	C14—C13—C16	120.4 (3)
O7—Zn1—N1	90.62 (7)	C13—C14—H14	120.1
O7—Zn1—N2^i^	91.96 (7)	C15—C14—C13	119.7 (2)
O7—Zn1—C1	122.20 (8)	C15—C14—H14	120.1
N1—Zn1—O1	86.95 (7)	C10—C15—H15	119.8
N1—Zn1—O2	90.42 (7)	C14—C15—C10	120.5 (2)
N1—Zn1—N2^i^	176.68 (7)	C14—C15—H15	119.8
N1—Zn1—C1	88.14 (7)	O6—C16—C13	124.9 (3)
N2^i^—Zn1—O2	86.32 (7)	O6—C16—H16	121 (2)
N2^i^—Zn1—C1	88.73 (7)	C13—C16—H16	114 (2)
O8—Zn2—O9	59.00 (7)	N1—C17—C18	121.8 (2)
O8—Zn2—C21	29.37 (7)	N1—C17—H17	119.1
O9—Zn2—C21	29.64 (7)	C18—C17—H17	119.1
O10—Zn2—O8	168.30 (8)	N2—C18—C17	121.7 (2)
O10—Zn2—O9	109.58 (8)	N2—C18—H18	119.2
O10—Zn2—O14	99.14 (8)	C17—C18—H18	119.2
O10—Zn2—N3	92.54 (8)	N2—C19—C20	122.0 (2)
O10—Zn2—N4^i^	90.13 (8)	N2—C19—H19	119.0
O10—Zn2—C21	139.14 (9)	C20—C19—H19	119.0
O14—Zn2—O8	92.03 (7)	N1—C20—C19	121.6 (2)
O14—Zn2—O9	150.78 (7)	N1—C20—H20	119.2
O14—Zn2—N3	92.41 (8)	C19—C20—H20	119.2
O14—Zn2—N4^i^	89.63 (8)	O8—C21—Zn2	59.97 (12)
O14—Zn2—C21	121.28 (8)	O8—C21—O9	121.1 (2)
N3—Zn2—O8	90.45 (7)	O8—C21—C22	119.2 (2)
N3—Zn2—O9	91.62 (7)	O9—C21—Zn2	61.18 (13)
N3—Zn2—N4^i^	176.35 (7)	O9—C21—C22	119.7 (2)
N3—Zn2—C21	91.63 (7)	C22—C21—Zn2	178.24 (17)
N4^i^—Zn2—O8	86.45 (7)	C23—C22—C21	120.9 (2)
N4^i^—Zn2—O9	85.14 (7)	C23—C22—C27	119.6 (2)
N4^i^—Zn2—C21	84.72 (7)	C27—C22—C21	119.5 (2)
C1—O1—Zn1	91.51 (15)	C22—C23—C24	119.5 (2)
C1—O2—Zn1	88.67 (15)	C22—C23—H23	120.2
C9—O3—Zn1	145.76 (18)	C24—C23—H23	120.2
Zn1—O7—H71	126.2 (19)	C23—C24—H24	119.7
Zn1—O7—H72	120.4 (18)	C25—C24—C23	120.7 (2)
H71—O7—H72	106 (2)	C25—C24—H24	119.7
C21—O8—Zn2	90.66 (15)	C24—C25—C26	119.9 (2)
C21—O9—Zn2	89.18 (15)	C24—C25—C28	119.3 (3)
C29—O10—Zn2	145.70 (18)	C26—C25—C28	120.8 (3)
Zn2—O14—H141	127 (2)	C25—C26—C27	119.5 (2)
Zn2—O14—H142	122 (2)	C25—C26—H26	120.2
H141—O14—H142	107 (3)	C27—C26—H26	120.2
C17—N1—Zn1	118.79 (16)	C22—C27—H27	119.6
C20—N1—Zn1	124.69 (16)	C26—C27—C22	120.8 (2)
C20—N1—C17	116.5 (2)	C26—C27—H27	119.6
C18—N2—Zn1^ii^	123.89 (16)	O12A—C28—O12B	114.5 (6)
C19—N2—Zn1^ii^	119.73 (16)	O12A—C28—C25	124.7 (4)
C19—N2—C18	116.3 (2)	O12A—C28—H28	90.8
C37—N3—Zn2	121.77 (16)	O12B—C28—C25	120.7 (6)
C40—N3—Zn2	122.19 (16)	O12B—C28—H28	90.8
C40—N3—C37	116.0 (2)	C25—C28—H28	90.8
C38—N4—Zn2^ii^	121.85 (16)	O10—C29—C30	115.4 (2)
C38—N4—C39	117.1 (2)	O11—C29—O10	126.5 (2)
C39—N4—Zn2^ii^	121.05 (16)	O11—C29—C30	118.1 (2)
O1—C1—Zn1	59.12 (12)	C31—C30—C29	120.3 (2)
O1—C1—O2	120.9 (2)	C31—C30—C35	119.9 (2)
O1—C1—C2	118.9 (2)	C35—C30—C29	119.8 (2)
O2—C1—Zn1	61.81 (13)	C30—C31—H31	120.3
O2—C1—C2	120.1 (2)	C32—C31—C30	119.5 (2)
C2—C1—Zn1	177.97 (18)	C32—C31—H31	120.3
C3—C2—C1	120.7 (2)	C31—C32—C33	120.6 (2)
C3—C2—C7	119.7 (2)	C31—C32—H32	119.7
C7—C2—C1	119.6 (2)	C33—C32—H32	119.7
C2—C3—C4	119.6 (2)	C32—C33—C34	119.8 (2)
C2—C3—H3	120.2	C32—C33—C36	119.5 (2)
C4—C3—H3	120.2	C34—C33—C36	120.7 (2)
C3—C4—H4	119.8	C33—C34—H34	120.0
C5—C4—C3	120.3 (3)	C35—C34—C33	119.9 (2)
C5—C4—H4	119.8	C35—C34—H34	120.0
C4—C5—C6	120.3 (2)	C30—C35—H35	119.9
C4—C5—C8	119.0 (3)	C34—C35—C30	120.2 (2)
C6—C5—C8	120.7 (3)	C34—C35—H35	119.9
C5—C6—C7	119.5 (3)	O13—C36—C33	125.7 (3)
C5—C6—H6	120.3	O13—C36—H36	120 (2)
C7—C6—H6	120.3	C33—C36—H36	114 (2)
C2—C7—H7	119.7	N3—C37—C38	121.9 (2)
C6—C7—C2	120.5 (2)	N3—C37—H37	119.0
C6—C7—H7	119.7	C38—C37—H37	119.0
O5A—C8—C5	125.8 (4)	N4—C38—C37	121.5 (2)
O5A—C8—H8	91.3	N4—C38—H38	119.2
O5B—C8—O5A	109.7 (8)	C37—C38—H38	119.2
O5B—C8—C5	124.3 (8)	N4—C39—C40	121.2 (2)
O5B—C8—H8	91.3	N4—C39—H39	119.4
C5—C8—H8	91.3	C40—C39—H39	119.4
O3—C9—C10	115.5 (2)	N3—C40—C39	122.2 (2)
O4—C9—O3	126.4 (2)	N3—C40—H40	118.9
O4—C9—C10	118.2 (2)	C39—C40—H40	118.9
			
O2—Zn1—O1—C1	0.71 (14)	Zn1—N1—C20—C19	179.19 (18)
O3—Zn1—O1—C1	1.0 (5)	C17—N1—C20—C19	−0.1 (4)
O7—Zn1—O1—C1	177.90 (15)	Zn1^ii^—N2—C18—C17	177.49 (19)
N1—Zn1—O1—C1	−91.63 (15)	C19—N2—C18—C17	−0.2 (4)
N2^i^—Zn1—O1—C1	85.90 (15)	Zn1^ii^—N2—C19—C20	−177.02 (19)
O1—Zn1—O2—C1	−0.71 (14)	C18—N2—C19—C20	0.8 (4)
O3—Zn1—O2—C1	179.35 (14)	Zn2—N3—C37—C38	−177.2 (2)
O7—Zn1—O2—C1	−6.6 (2)	C40—N3—C37—C38	0.2 (4)
N1—Zn1—O2—C1	85.47 (15)	Zn2—N3—C40—C39	176.65 (19)
N2^i^—Zn1—O2—C1	−93.92 (15)	C37—N3—C40—C39	−0.7 (4)
O1—Zn1—O3—C9	−20.6 (6)	Zn2^ii^—N4—C38—C37	178.53 (19)
O2—Zn1—O3—C9	−20.4 (3)	C39—N4—C38—C37	−0.8 (4)
O7—Zn1—O3—C9	162.5 (3)	Zn2^ii^—N4—C39—C40	−179.05 (19)
N1—Zn1—O3—C9	71.3 (3)	C38—N4—C39—C40	0.3 (4)
N2^i^—Zn1—O3—C9	−105.8 (3)	C3—C2—C1—O1	165.0 (2)
C1—Zn1—O3—C9	−19.9 (4)	C7—C2—C1—O1	−13.2 (3)
O1—Zn1—N1—C17	−55.30 (18)	C3—C2—C1—O2	−13.1 (4)
O1—Zn1—N1—C20	125.40 (19)	C7—C2—C1—O2	168.7 (2)
O2—Zn1—N1—C17	−114.10 (18)	C1—C2—C3—C4	−176.2 (2)
O2—Zn1—N1—C20	66.59 (19)	C7—C2—C3—C4	2.0 (4)
O3—Zn1—N1—C17	135.70 (18)	C1—C2—C7—C6	177.4 (2)
O3—Zn1—N1—C20	−43.6 (2)	C3—C2—C7—C6	−0.8 (4)
O7—Zn1—N1—C17	37.53 (18)	C2—C3—C4—C5	−1.4 (4)
O7—Zn1—N1—C20	−141.78 (19)	C6—C5—C4—C3	−0.4 (4)
C1—Zn1—N1—C17	−84.67 (18)	C8—C5—C4—C3	−178.9 (3)
C1—Zn1—N1—C20	96.02 (19)	C4—C5—C6—C7	1.6 (4)
O1—Zn1—C1—O2	178.8 (2)	C8—C5—C6—C7	−179.9 (3)
O2—Zn1—C1—O1	−178.8 (2)	C4—C5—C8—O5A	−177.0 (4)
O3—Zn1—C1—O1	−179.71 (14)	C4—C5—C8—O5B	8.1 (11)
O3—Zn1—C1—O2	−0.9 (2)	C6—C5—C8—O5A	4.4 (6)
O7—Zn1—C1—O1	−2.48 (17)	C6—C5—C8—O5B	−170.5 (10)
O7—Zn1—C1—O2	176.29 (13)	C2—C7—C6—C5	−1.0 (4)
N1—Zn1—C1—O1	87.09 (15)	C11—C10—C9—O3	−11.0 (3)
N1—Zn1—C1—O2	−94.15 (15)	C11—C10—C9—O4	170.3 (3)
N2^i^—Zn1—C1—O1	−93.99 (15)	C15—C10—C9—O3	167.2 (2)
N2^i^—Zn1—C1—O2	84.77 (15)	C15—C10—C9—O4	−11.5 (4)
O9—Zn2—O8—C21	−0.91 (14)	C9—C10—C15—C14	−177.4 (2)
O10—Zn2—O8—C21	12.3 (5)	C11—C10—C15—C14	0.8 (4)
O14—Zn2—O8—C21	175.05 (15)	C12—C11—C10—C9	179.2 (2)
N3—Zn2—O8—C21	−92.53 (15)	C12—C11—C10—C15	1.0 (4)
N4^i^—Zn2—O8—C21	85.54 (15)	C10—C11—C12—C13	−1.9 (4)
O8—Zn2—O9—C21	0.90 (14)	C11—C12—C13—C14	1.0 (4)
O10—Zn2—O9—C21	−176.27 (14)	C11—C12—C13—C16	−178.7 (3)
O14—Zn2—O9—C21	−7.4 (2)	C12—C13—C16—O6	172.9 (3)
N3—Zn2—O9—C21	90.46 (15)	C14—C13—C16—O6	−6.8 (5)
N4^i^—Zn2—O9—C21	−87.87 (15)	C15—C14—C13—C12	0.8 (4)
O8—Zn2—O10—C29	−37.1 (6)	C15—C14—C13—C16	−179.4 (3)
O9—Zn2—O10—C29	−25.1 (4)	C10—C15—C14—C13	−1.7 (4)
O14—Zn2—O10—C29	160.4 (3)	N2—C18—C17—N1	−0.5 (4)
N3—Zn2—O10—C29	67.6 (3)	N1—C20—C19—N2	−0.6 (4)
N4^i^—Zn2—O10—C29	−109.9 (3)	O8—C21—C22—C23	−175.8 (2)
C21—Zn2—O10—C29	−27.9 (4)	O9—C21—C22—C23	4.4 (4)
O8—Zn2—N3—C37	−56.67 (19)	O8—C21—C22—C27	2.6 (3)
O8—Zn2—N3—C40	126.14 (18)	O9—C21—C22—C27	−177.2 (2)
O9—Zn2—N3—C37	−115.67 (18)	C21—C22—C23—C24	177.7 (2)
O9—Zn2—N3—C40	67.14 (19)	C27—C22—C23—C24	−0.7 (4)
O10—Zn2—N3—C37	134.64 (19)	C21—C22—C27—C26	−176.3 (2)
O10—Zn2—N3—C40	−42.54 (19)	C23—C22—C27—C26	2.1 (4)
O14—Zn2—N3—C37	35.38 (19)	C22—C23—C24—C25	−1.5 (4)
O14—Zn2—N3—C40	−141.80 (19)	C26—C25—C24—C23	2.4 (4)
C21—Zn2—N3—C37	−86.02 (19)	C28—C25—C24—C23	−175.7 (3)
C21—Zn2—N3—C40	96.79 (19)	C24—C25—C28—O12A	173.7 (4)
O8—Zn2—C21—O9	−178.4 (2)	C24—C25—C28—O12B	−3.4 (8)
O9—Zn2—C21—O8	178.4 (2)	C26—C25—C28—O12A	−4.3 (6)
O10—Zn2—C21—O8	−176.21 (15)	C26—C25—C28—O12B	178.6 (7)
O10—Zn2—C21—O9	5.4 (2)	C27—C26—C25—C24	−1.0 (4)
O14—Zn2—C21—O8	−5.79 (18)	C27—C26—C25—C28	177.1 (3)
O14—Zn2—C21—O9	175.79 (14)	C22—C27—C26—C25	−1.3 (4)
N3—Zn2—C21—O8	88.02 (15)	C31—C30—C29—O10	−9.5 (3)
N3—Zn2—C21—O9	−90.40 (15)	C31—C30—C29—O11	172.2 (3)
N4^i^—Zn2—C21—O8	−92.14 (15)	C35—C30—C29—O10	170.5 (2)
N4^i^—Zn2—C21—O9	89.44 (15)	C35—C30—C29—O11	−7.8 (4)
Zn1—O1—C1—O2	−1.3 (2)	C29—C30—C35—C34	−179.3 (2)
Zn1—O1—C1—C2	−179.36 (19)	C31—C30—C35—C34	0.7 (4)
Zn1—O2—C1—O1	1.2 (2)	C32—C31—C30—C29	−179.3 (2)
Zn1—O2—C1—C2	179.30 (19)	C32—C31—C30—C35	0.7 (4)
Zn1—O3—C9—O4	−3.0 (5)	C30—C31—C32—C33	−1.7 (4)
Zn1—O3—C9—C10	178.4 (2)	C31—C32—C33—C34	1.3 (4)
Zn2—O8—C21—O9	1.6 (2)	C31—C32—C33—C36	−178.9 (3)
Zn2—O8—C21—C22	−178.21 (19)	C32—C33—C36—O13	172.4 (3)
Zn2—O9—C21—O8	−1.6 (2)	C34—C33—C36—O13	−7.7 (5)
Zn2—O9—C21—C22	178.23 (19)	C35—C34—C33—C32	0.1 (4)
Zn2—O10—C29—O11	6.8 (5)	C35—C34—C33—C36	−179.7 (3)
Zn2—O10—C29—C30	−171.3 (2)	C33—C34—C35—C30	−1.1 (4)
Zn1—N1—C17—C18	−178.7 (2)	N4—C38—C37—N3	0.6 (4)
C20—N1—C17—C18	0.7 (4)	N3—C40—C39—N4	0.5 (4)

Symmetry codes: (i) *x*, *y*−1, *z*; (ii) *x*, *y*+1, *z*.

### Hydrogen-bond geometry (Å, º)

Cg8 and Cg10 are the centroids of rings *B* (C10–C15) and *E* (C30–C35), respectively.

**Table:**

*D*—H···*A*	*D*—H	H···*A*	*D*···*A*	*D*—H···*A*
O7—H71···O9	0.90 (2)	1.82 (2)	2.694 (3)	165 (2)
O7—H72···O11	0.87 (2)	1.78 (2)	2.640 (3)	170 (2)
O14—H141···O2^iii^	0.83 (2)	1.90 (2)	2.705 (3)	165 (2)
O14—H142···O4^iii^	0.84 (2)	1.80 (3)	2.635 (3)	172 (3)
C17—H17···O12*A*^iv^	0.93	2.56	3.375 (5)	146
C19—H19···O6^v^	0.93	2.47	3.222 (4)	138
C23—H23···O1	0.93	2.57	3.361 (3)	143
C38—H38···O5*A*^iv^	0.93	2.59	3.381 (4)	144
C39—H39···O13^vi^	0.93	2.47	3.154 (4)	130
C12—H12···*Cg*10^vii^	0.93	2.81	3.579 (3)	140
C32—H32···*Cg*8^vii^	0.93	2.78	3.468 (3)	132

Symmetry codes: (iii) *x*, −*y*+3/2, *z*−1/2; (iv) −*x*+1, *y*+1/2, −*z*+1/2; (v) −*x*+2, −*y*+2, −*z*+1; (vi) −*x*+2, *y*+1/2, −*z*+1/2; (vii) −*x*, *y*−1/2, −*z*+1/2.
